# Persistence of Zika Virus in Breast Milk after Infection in Late Stage of Pregnancy

**DOI:** 10.3201/eid2305.161538

**Published:** 2017-05

**Authors:** José R. Sotelo, Andre B. Sotelo, Fabio J.B. Sotelo, André M. Doi, Joao R.R. Pinho, Rita de Cassia Oliveira, Alanna M.P.S. Bezerra, Alice D. Deutsch, Lucy S. Villas-Boas, Alvina C. Felix, Camila M. Romano, Clarisse M. Machado, Maria C.J. Mendes-Correa, Rubia A.F. Santana, Fernando G. Menezes, Cristovao L.P. Mangueira

**Affiliations:** Sotelo Clinic, São Paulo, (J.R. Sotelo, A.B. Sotelo, F.J.B. Sotelo);; Hospital Israelita Albert Einstein, São Paulo, Brazil (J.R. Sotelo, A.B. Sotelo, F.J.B. Sotelo, A.M. Doi, J.R.R. Pinho, R. de Cassia Oliveira, A.M.P.S. Bezerra, A.D. Deutsch, R.A.F. Santana, F.G. Menezes, C.L.P. Mangueira);; University of São Paulo, São Paulo (J.R.R. Pinho, L.S. Villas-Boas, A.C. Felix, C.M. Romano, C.M. Machado, Maria C.J. Mendes-Correa)

**Keywords:** Zika virus, viruses, infection, persistence, pregnancy, late stage, fetus, newborn, breast milk, colostrum, mosquitoes, zoonoses, Brazil

## Abstract

We detected Zika virus in breast milk of a woman in Brazil infected with the virus during the 36th week of pregnancy. Virus was detected 33 days after onset of signs and symptoms and 9 days after delivery. No abnormalities were found during fetal assessment or after birth of the infant.

Zika virus belongs to the family *Flaviviridae* and was first described in 1947. The first outbreak of infection with this virus was on Yap Island, Micronesia, in 2007 (*1*). The largest outbreak was in French Polynesia in 2013 (*2*).

The first cases of infection in Brazil were reported in Bahia State in 2015. Zika virus has since spread to >14 states in Brazil. Recently, the World Health Organization concluded that Zika virus is a cause of congenital brain abnormalities, including microcephaly; growth restriction and other damage, such as ophthalmologic alterations, also have been observed in neonates (*3*–*7*). We report a case of Zika virus infection in Brazil in an advanced stage of pregnancy and persistence of virus in breast milk 33 days after onset of signs and symptoms and 9 days after delivery.

A 28-year-old pregnant woman in the 36th week of gestation and living in Manaus, Brazil, reported mosquito bites and local infestation by *Aedes aegypti* mosquitoes in her neighborhood. She became ill and had a low-grade fever (temperature 38°C), rash (Figure), myalgia, and joint pain in the hands and wrists. PCR of blood samples showed a positive result for Zika virus (*8*). On the 4th day after illness onset, her clinical symptoms worsened, and she went to São Paulo, Brazil, for clinical evaluation. A timeline of symptoms and results of radiographic and laboratory studies is shown in the Figure.

**Figure:**
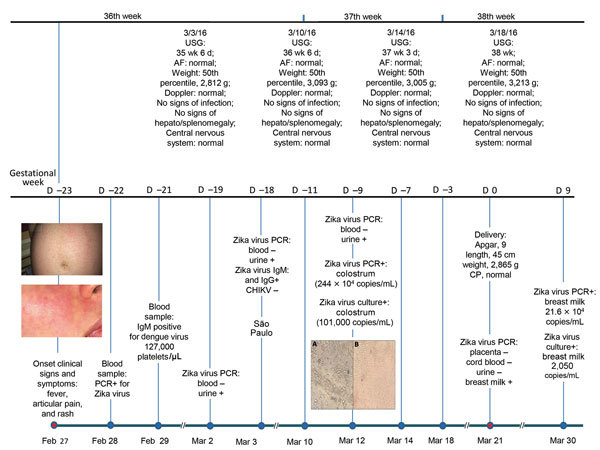
Timeline and clinical findings for a 28-year-old woman in the 36th week of pregnancy who had persistence of Zika virus in breast milk after infection in late stage of pregnancy and for her newborn, Manaus, Brazil. Top: ultrasound result for mother, fetus, and newborn. Bottom: follow-up test results for mother, fetus, and newborn. The 2 panels on the left show abdominal (top) and facial (bottom) rashes on the mother at the time of illness onset. Middle panels: Vero cell culture of breast milk and colostrum. A) Cells not infected with Zika virus. Original magnification ×10. B) Cells infected with Zika virus. Original magnification ×10. AF, amniotic fluid; CHIKV, chikungunya virus; CP, cranial perimeter; USG, ultrasound guidance.

General examinations were requested, and a PCR for Zika virus was repeated for blood and urine samples. Virus was detected only in urine. Serologic analysis detected dengue virus IgM and IgG; no antibodies against nonstructural protein 1 of this virus were detected. Results of reverse transcription PCR (RT-PCR) were negative for chikungunya virus. These findings were compatible with acute or recent Zika virus infection.

Fetal assessment was performed by using morphologic ultrasound at 35, 36, 37, and 38 weeks of gestation. We found no evidence of growth restriction, microcephaly, or cerebral calcifications. On the 22nd day after illness onset, blood and urine samples were tested by RT-PCR for Zika virus; results were negative. However, a colostrum sample was tested by RT-PCR and contained Zika virus (244 × 10^4^ copies/mL) (*8*).

The baby was delivered during the 38th gestational week and had Apgar scores of 9 at 1 minute and 10 at 5 minutes. Birthweight was 2,860 g, and the newborn had a normal cranial circumference. RT-PCR for Zika virus was performed for amniotic fluid, umbilical cord blood, and placenta samples; results were negative. A urine sample from the newborn also showed a negative result for virus. However, breast milk remained positive for Zika virus.

Analysis of the placenta showed maturation compatible with the third trimester of gestation, preservation of chorioamniotic membranes, and no signs of infection or malignancy. No viral inclusions were observed. After birth, the mother and baby remained in good clinical condition and showed no signs or symptoms of infection. Breast-feeding was not recommended because of persistence of virus detected by PCR in breast milk. The mother and baby were discharged 2 days after birth.

We performed viral culture on Vero cells of breast milk and colostrum samples (Figure). A cytopathic effect was observed, which demonstrated viability and infectivity of the virus.

The most recent RT-PCR for Zika virus was performed for breast milk 33 days after onset of signs and symptoms and 9 days after delivery. RT-PCR results remained positive, and a high virus load (216,000 copies/mL) was observed. The mother and the medical team supported a decision to avoid breast-feeding once RT-PCR confirmed presence of the virus.

No studies have confirmed Zika virus transmission by breast-feeding or provided knowledge about the pathophysiology of infection. Our report describes a case of Zika virus infection in a patient at 36 weeks of pregnancy. The patient and baby remained well after delivery, with no evidence of transmission of Zika virus to the newborn. However, we detected persistence of virus by RT-PCR in breast milk from samples during the pregnancy (in colostrum) 33 days after onset of signs and symptoms (in breast milk).

Two studies have reported Zika virus in breast-feeding−related fluids. One study reported a virus load of 2.9 × 10^4^ copies/mL by RT-PCR but no replicative virus in culture (*9*). A second study reported a virus load of 8.5 × 10^4^ copies/mL and infective viral particles 3 days after birth (*10*).

We detected Zika virus in colostrum (2.44 × 10^6^ copies/mL) and breast milk 9 days after birth (216,000 copies/mL) by PCR. We also observed cytopathic effect in virus culture, which showed infectivity of the virus. Our data provide evidence that Zika virus can persist in some tissues for a long period. Moreover, viral culture showed potential infectivity of the virus.

The World Health Organization does not recommend that mothers avoid breast-feeding in cases such as the one mentioned in this report. However, with indications that virus might be present and persistent in breast milk, further studies should be performed to elucidate the potential transmission of Zika virus to the newborn.
